# Retroperitoneal Ganglioneuroma (GN): Case report in 14 years old boy

**DOI:** 10.1016/j.ijscr.2019.06.011

**Published:** 2019-06-12

**Authors:** Nahlah Arab, Ashwag Alharbi

**Affiliations:** Department of General Surgery, Prince Sultan Military Medical City, Riyadh, Saudi Arabia

**Keywords:** Ganglioneuroma (GN), Inferior vena cava (IVC), Computed tomography (CT), Magnetic resonance imaging (MRI)

## Abstract

**Introduction:**

The ganglioneuroma is a rare tumor arising from sympathetic nervous system, which is composed of gangliocytes and mature stroma.

**Presentation of the case:**

A retroperitoneal ganglioneuroma was found in a 14 years old Saudi boy, presented with recurrent vomiting. CT image showing a retroperitoneal cyst near the inferior vena cava and right iliac vessels. Treated by laparoscopic cystic excision, histopathology of the cyst showing ganglioneuroma, completely excised.

**Discussion:**

Ganglioneuromas arise from the sympathetic chain, The most common sites of presentation are the posterior mediastinum, retroperitoneum, head and neck region. GN are usually asymptomatic found incidentally on abdominal imaging. The treatment of retroperitoneal mass is complete surgical excision.

**Conclusion:**

Ganglioneuroma (GN) is a rare benign tumor, usually asymptomatic. One of the most common site of presentation is retroperitoneum, the main treatment for that is complete surgical excision.

## Highlights

• Ganglioneuroma (GN) is a rare benign tumor.
• GN are usually asymptomatic found incidentally on abdominal imaging or having nonspecific symptoms related to mass effect.
• The cornerstone for the management its remain on complete surgical excision.

## 1 Introduction

The work has been reported in line with the SCARE criteria [8].

Neuroblastic tumors are the most common extra-cranial solid tumors in childhood. They arise from the neural crest include neuroblastoma, ganglioneuroblastoma (nodular or intermixed), and ganglioneuroma [1].

The most benign tumor is the ganglioneuroma, which is composed of gangliocytes and mature stroma. Ganglioneuroblastoma is composed of both mature gangliocytes and immature neuroblasts and has intermediate malignant potential. Neuroblastoma is the most immature, undifferentiated, and malignant tumor of the three [2].

## 2 Presentation of case

In January 2019, A 14 years old Saudi boy not known to have any medical illness, presented to the clinic complained of recurrent vomiting for the last 2 months. The vomiting was food content. No history of abdominal pain, nausea, change on bowel habit, weight loss and fever. No family history of any malignancy nor history of radiotherapy or smoking.

On examination: Conscious oriented alert, not on respiratory distress, not pallor neither cyanosis, he looks underweight. Systemic examination unremarkable. Cardiovascular and chest examination were unremarkable. Abdominal examination swelling was notice at epigastric and right upper quadrant area, abdomen was soft, with no tenderness, normal bowel sound.

**Fig. 1:**
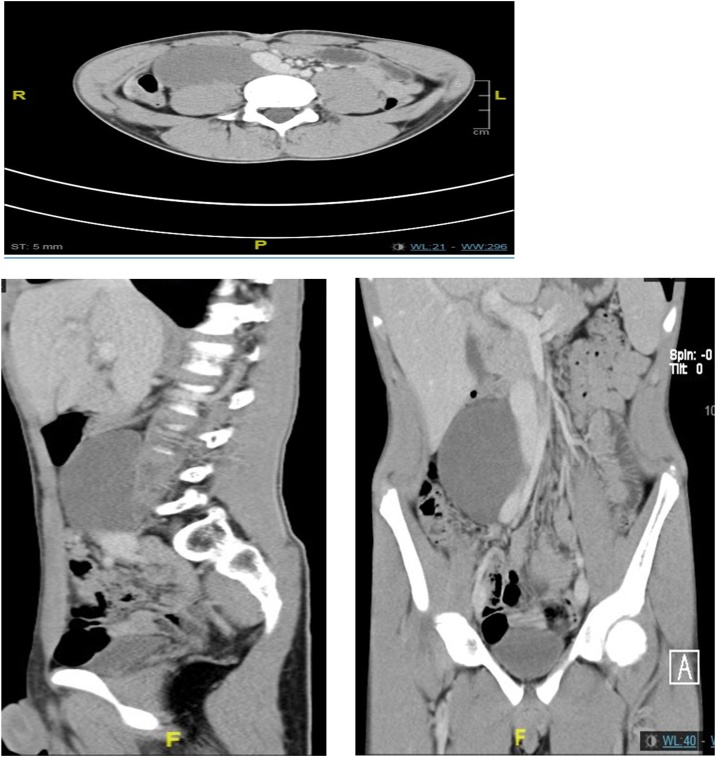
CT abdomen showing a cystic lesion at lower right retroperitoneal space, near the inferior vena cava and right iliac vessels.

His workup wbc 7 × 10^9^/l hgb12.9 g/dl platelets 272 × 10^9^/l, albumin 47 g/l. Abd x-ray reveled normal distribution of bowel gas. CT abdomen Fig. 1 reveled a cystic lesion at lower right retroperitoneal space without septation, calcification, fatty or solid component, its abutting medially the lower inferior vena cava and right iliac vessels, anteriorly displacing the peritoneal space and abutting the anterior abdominal wall muscle, laterally abutting the ascending colon and posteriorly abutting the psoas muscle. Measures about 4.1cm*6.5 cm*8.5 cm.

**Fig. 2:**
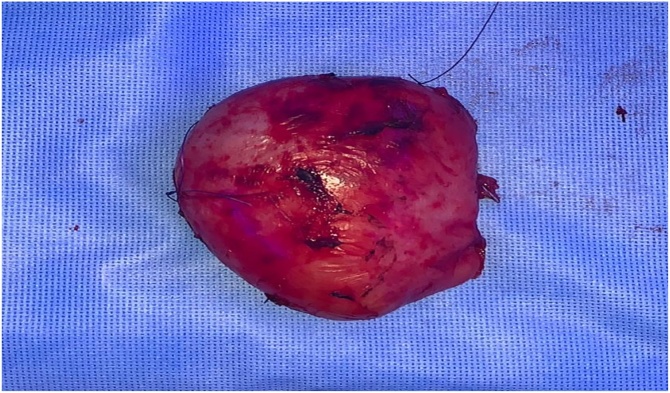
Cystic mass about 10* 9*6 cm.

Surgical management was considered for him including: laparoscopic exploration with cystic excision, patient was on supine position. Intraoperative finding was showing encapsulated mass measured 10 × 9 × 6 cm in size adherent to the IVC, its was carefully dissected from it using blunt dissection and electrocautery Fig. 2.

Histopathology of the cyst showing ganglioneuroma, completely excised, composed of numerous spindle cells consistent with shawanian cell mixed with ganglion cells, no neuroblastic component. Patient was discharge on good health condition during his visit on the clinic, improve of his complain for regular follow up.

## 3 Discussion

Ganglioneuroma (GN) is a rare benign tumor, its either happen spontaneously or during the therapy for neuroblastoma with either chemotherapy or radiation therapy. Association with Turner’s syndrome and multiple endocrine neoplasia II has been reported [3].

Ganglioneuromas arise from the sympathetic chain. The most common sites of presentation are the posterior mediastinum, retroperitoneum, head and neck region [4].

GN are usually asymptomatic found incidentally on abdominal imaging or having nonspecific symptoms related to mass effect and compression of surrounding organs.

Back pain due to spinal deformity, vomiting and abdominal pain from compressing the stomach or bowel. The compression of the diaphragm muscle patients exhibited dyspnea. Changes in gait, weakness of the muscles and senses, from the mass compresses the spinal cord. Some patient may have Hypertension and diarrhea if the tumor secretes catecholamine and vasoactive intestinal peptide [5].

The imaging modality of choice is contrast enhanced CT scan and MRI imaging. For identifying the relationship of the tumor with surrounding organs and vital structures. The ganglioneuromas appear as an oval, well-defined mass in the adrenal gland or in extra-adrenal location with low attenuation on unenhanced CT scans, homogeneous low signal intensity on T1-weighted images and homogeneous high signal intensity on T2-weighted image on MRI. The definitive diagnosis is made by the histopathology [6].

The treatment of retroperitoneal mass is complete surgical excision. One of the limitation factor for complete excision the tumor surrounding vital organs and blood vessels. The surgical resection of these tumors can be undertaken via laparoscopic or open technique. Open surgery preferred while operating on tumor in close proximity of or surrounding important blood vessels in the abdominal cavity [6].

Follow-up is needed the tumor have slow progression potential and late recurrence [7].

## 4 Conclusion

Ganglioneuroma (GN) is a rare benign tumor, usually asymptomatic. One of the most common site of presentation is retroperitoneum, the main treatment for that is complete surgical excision. Regular follow-up is needed due to late recurrence.

## Declaration of Competing Interest

All of authors have no conflict of interest.

## Funding

This research did not receive any specific grant from funding agencies in the public, commercial, or not-for-profit sectors.

## Ethical Approval

In our institute, ethical approval is exempted, depend on acquired patient consent.

## Consent

Written informed consent was obtained from the guardian on behalf of the patient for publication of this case report. A copy of the written consent is available for review by the Editor-in-Chief of this journal.

## Author contribution

Nahlah arab and Ashwag alharbi contributed to manuscript preparation, manuscript editing, manuscript review.

## Registration of Research Studies

We don’t need to register this work.

## Guarantor

The Guarantor is DR. Ashwag Alharbi.

## Provenance and peer review

Not commissioned, externally peer-reviewed.
